# Dual effects of fasting on gut microbiota and metabolism: protective adaptation versus barrier dysfunction risks

**DOI:** 10.3389/fnut.2026.1895190

**Published:** 2026-08-17

**Authors:** Jifei Miao, Zhiping Wang, Xianjie Chen, Jun Zhong, Sen Ye

**Affiliations:** 1 Shenzhen Baoan Chinese Medicine Hospital, Guangzhou University of Chinese Medicine, Shenzhen, Guang Dong, China; 2 State Key Laboratory of Traditional Chinese Medicine Syndrome, Guangzhou University of Chinese Medicine, Guangzhou, China; 3 Key Laboratory of Tropical Translational Medicine of Ministry of Education, School of Basic Medicine and Life Sciences, Hainan Medical University, Haikou, China

**Keywords:** *Akkermansia*, *Erysipelatoclostridium*, fasting, gut microbiota, inflammation, intestinal barrier, metabolites

## Abstract

**Introduction:**

Fasting reshapes gut microbiota and metabolism, but its time-dependent dual impacts on intestinal barrier remain unclear. This study explored how 12 h and 24 h acute fasting modulate gut microbes, metabolites, epithelial structure and inflammatory resilience in mice.

**Methods:**

C57BL/6 J mice were assigned to ad libitum feeding, 12 h fasting and 24 h fasting groups. We integrated PacBio 16S rRNA sequencing, untargeted LC-MS metabolomics, colon/cecum histology, Occludin immunofluorescence and CLP sepsis model, with LEfSe, multivariate statistics and correlation analysis for data interpretation. Data are available via MetaboLights with identifier MTBLS15098.

**Results:**

Fasting induced time-dependent microbial and metabolic remodeling: *Akkermansia muciniphila* peaked at 12 h fasting, while prolonged fasting elevated microbial richness and inflammation-associated taxa. Shared fasting-responsive metabolites altered pathways of immunity and energy metabolism. Both fasting durations reduced crypt depth and Occludin expression, with 24 h fasting triggering worse epithelial damage and higher post-CLP mortality. Correlations linked mucosal-protective microbes to beneficial metabolites and pro-inflammatory taxa to lipid mediators.

**Discussion:**

Short-term fasting generates favorable microbial-metabolic adaptation yet initiates mild epithelial injury, while 24 h fasting aggravates barrier defects and weakens anti-inflammatory resilience. Beneficial microbiota signatures are decoupled from intact mucosa, indicating fasting duration determines the balance between gut adaptation and epithelial vulnerability.

## 1 Introduction

Fasting is increasingly recognized as a beneficial intervention for improving metabolic health, modulating immune function, and extending lifespan. Emerging evidence highlights that controlled periods of fasting not only influence host energy metabolism but also profoundly reshape the composition and functionality of the gut microbiota (1, 2). These microbial communities play critical roles in regulating host metabolic homeostasis, gut barrier integrity, immune modulation, and systemic inflammatory responses (3). Despite growing interest in fasting as a therapeutic approach, the precise microbial and metabolic adaptations induced by varying durations of fasting remain incompletely understood.

A crucial element of gut homeostasis is the intestinal barrier, a selectively permeable structure that regulates nutrient absorption, prevents pathogen entry, and maintains mucosal immune balance. Disruption of this barrier, characterized by increased intestinal permeability, contributes to systemic inflammation and the pathogenesis of numerous gastrointestinal disorders such as inflammatory bowel disease (IBD) and irritable bowel syndrome (IBS) (4). Conversely, strategies that reinforce barrier integrity can exert protective effects, alleviating disease severity and enhancing metabolic resilience (5, 6). Recent studies indicate that fasting might have dual effects on gut barrier function: while short-duration fasting promotes gut epithelial renewal and barrier protection, long-duration or excessively stringent fasting regimens may induce intestinal stress, exacerbating inflammation and barrier disruption (7, 8).

Among the microbiota modulated by fasting, certain taxa such as *Akkermansia muciniphila* have garnered considerable attention due to their positive roles in gut barrier function and metabolic health. Accumulating evidence indicates that dietary interventions, including fasting, significantly enhance the abundance of *Akkermansia*, correlating with improved mucus layer integrity, enhanced barrier function, and better metabolic profiles (6, 9, 10). In contrast, the fasting-induced expansion of potentially pro-inflammatory microbial taxa, such as *Erysipelatoclostridium*, raises concerns regarding adverse inflammatory outcomes. Elevated levels of inflammatory mediators, including prostaglandins, observed under certain fasting conditions highlight a potential downside to long-duration fasting regimens, especially in susceptible individuals (11, 12). Such microbial changes underline the complexity and nuanced nature of fasting interventions, emphasizing the need for careful delineation of fasting durations and personalized approaches.

Furthermore, fasting-induced shifts in gut microbiota composition are accompanied by significant alterations in host metabolomic profiles, encompassing pathways critical for energy regulation, neurotransmitter function, inflammation, and gut-brain axis signaling. Metabolites play pivotal roles in mediating interactions between microbiota and host physiology, influencing both intestinal and systemic health outcomes (13–15). Understanding how these metabolites fluctuate under different fasting conditions, and their specific associations with distinct microbial taxa, could offer valuable insights into optimizing fasting strategies.

Therefore, the present study aimed to systematically investigate how different acute fasting durations reshape gut microbial composition, host metabolite profiles, intestinal epithelial morphology, and susceptibility to inflammatory stress. We focused on 12 h and 24 h fasting because these time points represent short-term overnight fasting and a more prolonged acute fasting condition, respectively. We hypothesized that fasting would induce duration-dependent microbial and metabolic adaptations, but that these changes might not necessarily parallel epithelial structural integrity. By integrating 16S rRNA gene sequencing, untargeted metabolomics, histological assessment, Occludin immunofluorescence, and a CLP inflammatory challenge model, this study sought to clarify whether fasting-associated microbial-metabolic remodeling is accompanied by preserved or impaired epithelial resilience.

## 2 Materials and methods

### 2.1 Animals and experimental design

Male C57BL/6 J mice (18–22 g, SPF grade; License No. SCXK (Yue) 20,180,034) were housed under standardized conditions (temperature 24 ± 3 °C, relative humidity 55 ± 5%, 12-h light/dark cycles), with free access to drinking water and rodent chow (60Co Mouse Diet 1,035, Seboina, China). Corncob bedding was also obtained from Seboina, China. All animal experimental procedures were performed in accordance with the guidelines approved by Guangzhou University of Chinese Medicine. (Approval Number: 20220119003).

Following 1 week of acclimatization, mice were randomly assigned to three experimental groups: the ad libitum feeding group (Food), the 12 h fasting group (Fast_12h), and the 24 h fasting group (Fast_24h). All fasting groups had continuous access to water. For 16S rRNA gene sequencing, untargeted metabolomic profiling, histological assessment, Occludin immunofluorescence staining, and related morphometric analyses, the same cohort of five mice per group was used. Intestinal contents were collected for microbiome and metabolomic analyses, whereas colon and cecum tissues from the same animals were processed for histological and immunofluorescence analyses. For the CLP survival experiment, an independent cohort of 12 mice per group was used. At the conclusion of the fasting intervention, mice designated for tissue collection were deeply anesthetized by inhalation of 3% isoflurane in 100% oxygen and subsequently euthanized by cervical dislocation. Death was confirmed by the absence of heartbeat and respiration before tissue collection. Colon segments were immediately isolated under sterile conditions, and intestinal contents were gently collected into sterile Eppendorf tubes, flash-frozen in liquid nitrogen, and stored at −80 °C for subsequent 16S rRNA gene sequencing and untargeted metabolomic analyses.

### 2.2 16S rRNA gene sequencing of intestinal contents

Microbial DNA extraction and subsequent sequencing analysis were performed by PANOMIX Biomedical Tech Co., Ltd. (Suzhou, China). Microbial genomic DNA was extracted from intestinal contents, quantified using a NanoDrop spectrophotometer, and assessed by 0.8% agarose gel electrophoresis. Full-length 16S rRNA gene amplicons were generated using a two-step PCR strategy with PacBio-compatible barcoded primers. PCR amplification was performed using Q5 high-fidelity DNA polymerase (NEB), and negative controls were included to monitor potential contamination. Amplicons were purified by 2% agarose gel electrophoresis, quantified using the Quant-iT PicoGreen dsDNA Assay Kit (Invitrogen), pooled according to sample-specific sequencing requirements, and sequenced on the PacBio Sequel platform.

Raw PacBio BAM files were processed using CCS software v4.0.0 to generate high-accuracy consensus reads, with the parameters––min-length 500 and––max-length 3,000. Reads were demultiplexed according to barcode sequences, barcode sequences were removed, and reverse-complement reads were converted to the forward orientation according to primer information. Sequence denoising was performed using a modified QIIME2-compatible DADA2 workflow for PacBio data. Briefly, primers were removed using qiime cutadapt trim-single, and quality filtering, denoising, and chimera removal were performed using qiime dada2 denoise-pacbio. The resulting amplicon sequence variants (ASVs) were merged across libraries, and singleton ASVs were removed. Therefore, ASVs rather than traditional OTUs were used for downstream analysis.

Taxonomic assignment was performed in QIIME2 using the classify-sklearn Naive Bayes classifier with default parameters against the Greengenes database, release 13.8. The ASV abundance table was rarefied to 95% of the minimum sample sequencing depth before diversity analyses. Alpha diversity indices, including Chao1, Simpson, Shannon, Pielou’s evenness, observed species, Faith’s phylogenetic diversity, and Good’s coverage, were calculated. Beta diversity was assessed using weighted UniFrac, unweighted UniFrac, and Bray-Curtis distances, followed by principal coordinate analysis (PCoA). Differential taxa were explored using LEfSe analysis with an LDA score threshold >2.0 and *p* < 0.05. LEfSe-derived taxa were interpreted as candidate discriminant taxa rather than validated biomarkers.

### 2.3 Untargeted metabolomic analysis

Untargeted metabolomic profiling of intestinal contents was performed by PANOMIX Biomedical Tech Co., Ltd. (Suzhou, China) using an LC–MS/MS-based workflow. Quality control (QC) samples were included during LC–MS detection to evaluate analytical stability and reproducibility. Raw mass spectrometry data were converted to mzXML format using MSConvert in ProteoWizard software package v3.0.8789. Peak detection, peak filtering, retention time correction, and peak alignment were performed using the XCMS package in R with the following parameters: bw = 2, ppm = 15, peakwidth = c(5, 30), mzwid = 0.015, mzdiff = 0.01, and method = “centWave.”

Metabolites were annotated based on accurate mass matching and MS/MS spectral matching against public and in-house databases, including the Human Metabolome Database (HMDB), MassBank, LipidMaps, mzCloud, Kyoto Encyclopedia of Genes and Genomes (KEGG), and an in-house metabolite database, with a mass tolerance of <30 ppm. Data normalization was performed using robust LOESS signal correction based on QC samples to reduce systematic variation. Features with relative standard deviations (RSDs) > 30% in QC samples were excluded from downstream analysis.

Multivariate analyses, including principal component analysis (PCA), partial least squares discriminant analysis (PLS-DA), and orthogonal partial least squares discriminant analysis (OPLS-DA), were performed using the R package ropls after data scaling. Model performance was evaluated using R^2^X, R^2^Y, and *Q*^2^ values, and permutation tests were used to assess potential model overfitting. Differential metabolites were screened using variable importance in projection (VIP), fold change (FC), *p*-values, and false discovery rate (FDR) values. FDR values were calculated using the Benjamini-Hochberg method. Metabolites with VIP > 1 and *p* < 0.05 were considered fasting-responsive metabolites, and FDR values were reported to assist interpretation of multiple-testing-adjusted significance.

### 2.4 Metabolic pathway analysis

Metabolite pathway enrichment analysis was performed using MetaboAnalyst (http://www.metaboanalyst.ca), which integrates enrichment analysis with pathway topology analysis. Significantly altered metabolites identified through metabolomics were mapped to KEGG metabolic pathways for comprehensive biological interpretation, visualized via KEGG Mapper tool.

### 2.5 Correlation analysis between gut microbiota and metabolites

Associations between fasting-responsive microbial taxa and differential metabolites were assessed using Pearson or Spearman correlation analysis depending on data distribution. Correlation coefficients and corresponding *p* values were calculated, and the results were visualized as heatmaps using R packages including pheatmap and corrplot. Correlations with *p* < 0.05 were considered statistically significant. These analyses were designed to identify microbiota-metabolite associations and were interpreted as hypothesis-generating rather than causal evidence.

### 2.6 Cecal ligation and puncture (CLP) model

To evaluate the impact of fasting on susceptibility to systemic inflammatory stress, a cecal ligation and puncture (CLP) model was employed. After the respective fasting interventions (food, 12 h fasting, or 24 h fasting), mice were anesthetized by inhalation of isoflurane (RWD Life Science, Shenzhen, China) delivered in 100% oxygen, using 3% isoflurane for induction and 1.5–2% isoflurane for maintenance throughout the procedure. Adequacy of anesthesia was confirmed by the loss of the pedal withdrawal reflex prior to surgery. A midline laparotomy was performed to expose the cecum. Approximately 50% of the cecum was ligated with a 5–0 silk suture without causing bowel obstruction, followed by a single puncture using a 21-gauge needle. A small amount of fecal content was extruded to ensure patency. The cecum was then returned to the peritoneal cavity, and the abdominal wall was closed with sutures. Mice were monitored for survival for up to 96 h after surgery.

### 2.7 Hematoxylin and eosin (H&E) staining

Colon and cecum tissues were fixed in 4% paraformaldehyde overnight at 4 °C, embedded in paraffin, and sectioned at a thickness of 4 μm. Tissue sections were deparaffinized in xylene, rehydrated through graded ethanol series, and subjected to hematoxylin and eosin staining according to standard protocols. Stained sections were examined under the Pannoramic Digital Scanner (3DHISTECH, Hungary), and histological parameters, including crypt (gland) depth, lymphoid follicle counts, and epithelial architecture, were quantitatively assessed using ImageJ software.

For gross colon length analysis, only intact colon samples suitable for standardized full-length measurement were included. Samples with mechanical disruption during tissue collection were excluded from this specific gross morphological endpoint because reliable full-length measurement was not possible. This exclusion applied only to colon length analysis and did not affect histological, immunofluorescence, microbiome, metabolomic, or CLP survival analyses.

For histological quantification, five mice per group were analyzed. For each mouse, five randomly selected non-overlapping microscopic fields were measured using ImageJ, and the mean value of these fields was used as the biological replicate for statistical analysis.

### 2.8 Immunofluorescence staining for tight junction proteins

To assess epithelial barrier integrity, immunofluorescence staining was performed to detect Occludin expression in colon tissues. After deparaffinization and rehydration, antigen retrieval was conducted by heating sections in 10 mM sodium citrate buffer (pH 6.0) at 95 °C for 20 min. Sections were blocked with 5% bovine serum albumin (BSA) for 1 h at room temperature and then incubated overnight at 4 °C with anti-Occludin primary antibody (1:200 dilution, abcam, England). After washing, sections were incubated with Alexa Fluor 594-conjugated secondary antibody (1:500 dilution) for 1 h at room temperature. Nuclei were counterstained with DAPI. Fluorescence images were captured using the Pannoramic Digital Scanner (3DHISTECH, Hungary).

### 2.9 Statistical analysis

Data are expressed as mean ± standard error of the mean (SEM). Comparisons among three groups were performed using one-way analysis of variance (ANOVA) followed by Tukey’s *post hoc* test when data met parametric assumptions. For non-parametric data, the Kruskal-Wallis test followed by appropriate post hoc comparisons was used. Alpha diversity indices were compared using the Kruskal-Wallis test. Beta diversity was visualized by PCoA based on weighted UniFrac, unweighted UniFrac, and Bray-Curtis distance matrices. Overall and pairwise differences in microbial community composition were assessed using PERMANOVA with 999 permutations. For pairwise comparisons, q values were used to account for multiple comparisons. For survival analysis after CLP surgery, Kaplan–Meier survival curves were generated, and statistical significance was assessed using the log-rank (Mantel-Cox) test. Microbiota-metabolite associations were evaluated using Pearson or Spearman correlation analysis, as appropriate. Differences were considered statistically significant at *p* < 0.05. Statistical analyses were performed using GraphPad Prism 9.0 and R software.

## 3 Results

### 3.1 Fasting alters gut microbiota diversity and structure

**Figure 1:**
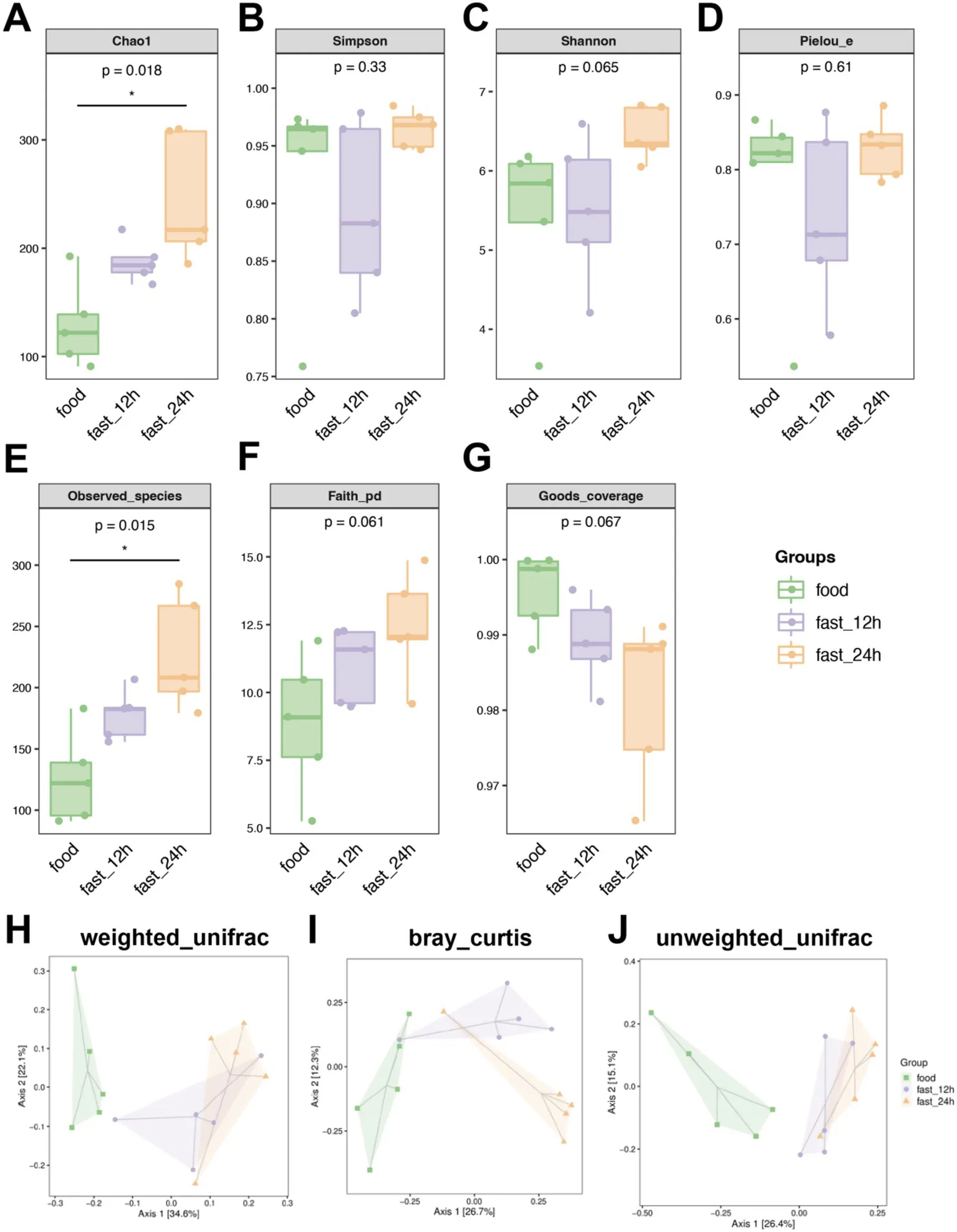
Fasting modulates gut microbiota diversity and structure. **(A–G)** Alpha diversity indices comparing microbial diversity across the three groups (*n* = 5). **(A)** Chao1 index. **(B)** Simpson index. **(C)** Shannon index. **(D)** Pielou’s evenness index. **(E)** Observed species index. **(F)** Faith’s phylogenetic diversity index. **(G)** Good’s coverage index. **(H)** PCoA using weighted UniFrac distances. **(I)** Bray–Curtis dissimilarity-based PCoA. **(J)** Unweighted UniFrac-based PCoA. PERMANOVA was used to assess overall and pairwise differences in beta diversity among groups, with detailed pseudo-*F*, *R*2, *P*, and *q* values provided in Supplementary Table S1.

Fasting significantly influenced the alpha diversity of the gut microbiota, as indicated by multiple diversity indices (Figures 1A–G). The Chao1 index, which estimates microbial richness, was significantly higher in the 24 h fasting group compared to the food group (*p* = 0.018), suggesting an increase in the total number of taxa with long-duration fasting (Figure 1A). Similarly, the observed species index showed a significant increase in the 24 h fasting group (*p* = 0.015), further supporting an expansion of microbial diversity under fasting conditions (Figure 1E).

In contrast, the Shannon diversity index exhibited a trend toward an increase in the 24 h fasting group but did not reach statistical significance (*p* = 0.065) (Figure 1C). Similarly, the Simpson index, which considers both richness and evenness, showed no significant differences among the groups (*p* = 0.33) (Figure 1B). Other indices, including Pielou’s evenness index (*p* = 0.61), Faith’s phylogenetic diversity (*p* = 0.061), and Good’s coverage index (*p* = 0.067), did not show statistically significant differences among the groups (Figure 1D,F,G).

Beta diversity analysis, which evaluates differences in overall microbial community composition, revealed distinct clustering patterns among the groups (Figures 1H–J). Principal coordinate analysis (PCoA) based on weighted UniFrac distances demonstrated a clear separation of the 24 h fasting group from the food group, indicating substantial shifts in microbial community structure with long-duration fasting (Figure 1H). A similar trend was observed in Bray–Curtis dissimilarity-based PCoA, which further supported fasting-induced microbial compositional changes (Figure 1I). Unweighted UniFrac analysis, which emphasizes the presence or absence of taxa rather than their relative abundances, also indicated fasting-dependent clustering, although with more variability (Figure 1J). PERMANOVA further supported significant differences in overall microbial community composition among the three groups based on weighted UniFrac (pseudo-*F* = 5.086, R^2^ = 0.459, *p* = 0.001), Bray-Curtis (pseudo-*F* = 2.596, R^2^ = 0.302, p = 0.001), and unweighted UniFrac (pseudo-*F* = 2.496, R^2^ = 0.294, p = 0.001) distance matrices. Pairwise PERMANOVA further showed that both 12 h fasting group and 24 h fasting group differed from the Food group across all three distance matrices. 12 h fasting group and 24 h fasting group also differed significantly based on weighted UniFrac and Bray-Curtis distances, but not based on unweighted UniFrac distance, suggesting that the duration-dependent difference between 12 h and 24 h fasting was more evident in abundance-weighted community structure than in presence/absence-based composition (Supplementary Table S1).

These findings suggest that long-duration fasting is associated with increased richness-related diversity indices and significant shifts in microbial community structure.

### 3.2 Fasting reshapes the composition and abundance of gut microbiota

**Figure 2:**
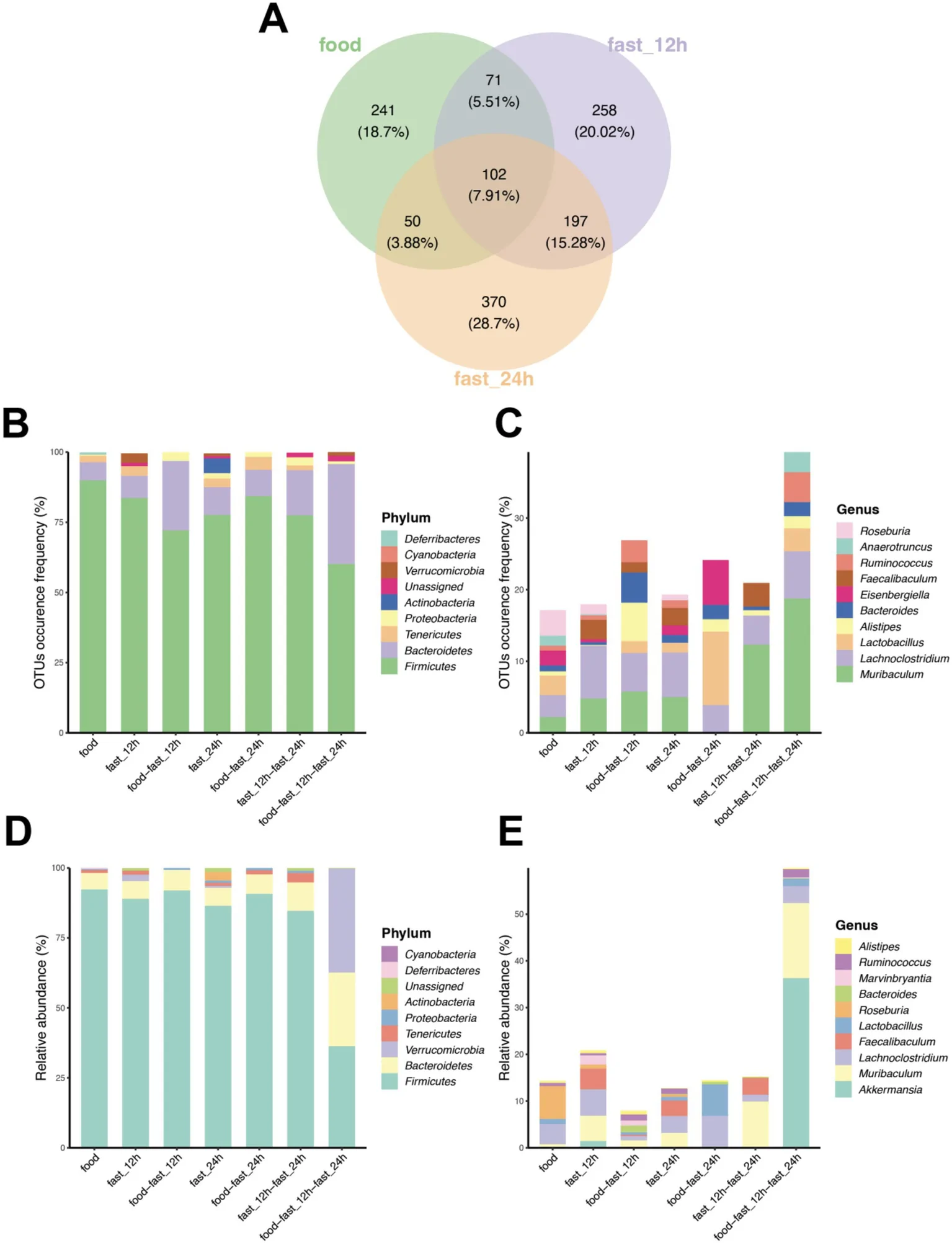
Fasting alters gut microbiota composition and abundance. **(A)** Venn diagram showing ASV distribution among food, 12 h fasting, and 24 h fasting groups. The number of unique and shared ASVs across groups indicates fasting-induced microbial shifts. **(B,C)** Occurrence frequency of different bacterial phyla **(B)** and genera **(C)** across groups. *Firmicutes* and *Bacteroidetes* remained dominant across all groups, while fasting led to increased prevalence of *Akkermansia*, *Faecalibaculum*, and *Lactobacillus*. **(D,E)** Relative abundance of bacterial phyla **(D)** and genera **(E)** across groups, illustrating fasting-induced compositional restructuring. *Akkermansia* was notably enriched in fasting groups, while *Muribaculaceae* showed a decreasing trend.

To further characterize the effects of fasting on gut microbial composition, we analyzed the occurrence and relative abundance of taxa across different fasting durations. The distribution of amplicon sequence variants (ASVs) showed substantial differences among the groups, with fasting leading to distinct shifts in microbial community structure (Figures 2A–E).

Analysis at the phylum level revealed that the dominant bacterial phyla remained consistent across all groups, primarily comprising *Firmicutes*, *Bacteroidetes*, *Verrucomicrobia*, and *Proteobacteria* (Figures 2B,D). However, the occurrence frequency and relative abundance of certain phyla varied in response to fasting. The relative abundance of *Firmicutes* decreased slightly in the 12 h and 24 h fasting groups, whereas Bacteroidetes and *Verrucomicrobia* showed an increasing trend in fasting conditions.

At the genus level, fasting significantly altered microbial composition, with notable changes in specific genera (Figures 2C,E). *Lactobacillus*, *Akkermansia*, and *Faecalibaculum* were more prevalent in fasting groups compared to the food group, whereas genera such as *Bacteroides*, *Alistipes*, and *Lachnoclostridium* exhibited variable patterns. Notably, *Akkermansia*, a mucin-degrading bacterium, was largely absent in the food group but became dominant in the fasting groups, particularly at 12 h of fasting. Conversely, *Muribaculaceae* was more abundant in the food group but showed a decreasing trend in fasting conditions.

The venn.abund.plot analysis (Figures 2D,E) further supported these findings by highlighting fasting-induced shifts in microbial abundance at both the phylum and genus levels. The restructuring of microbial communities with long-duration fasting suggests that microbial adaptation to nutrient deprivation may play a role in gut homeostasis and host metabolism.

### 3.3 Fasting induces specific alterations in gut microbiota composition

**Figure 3:**
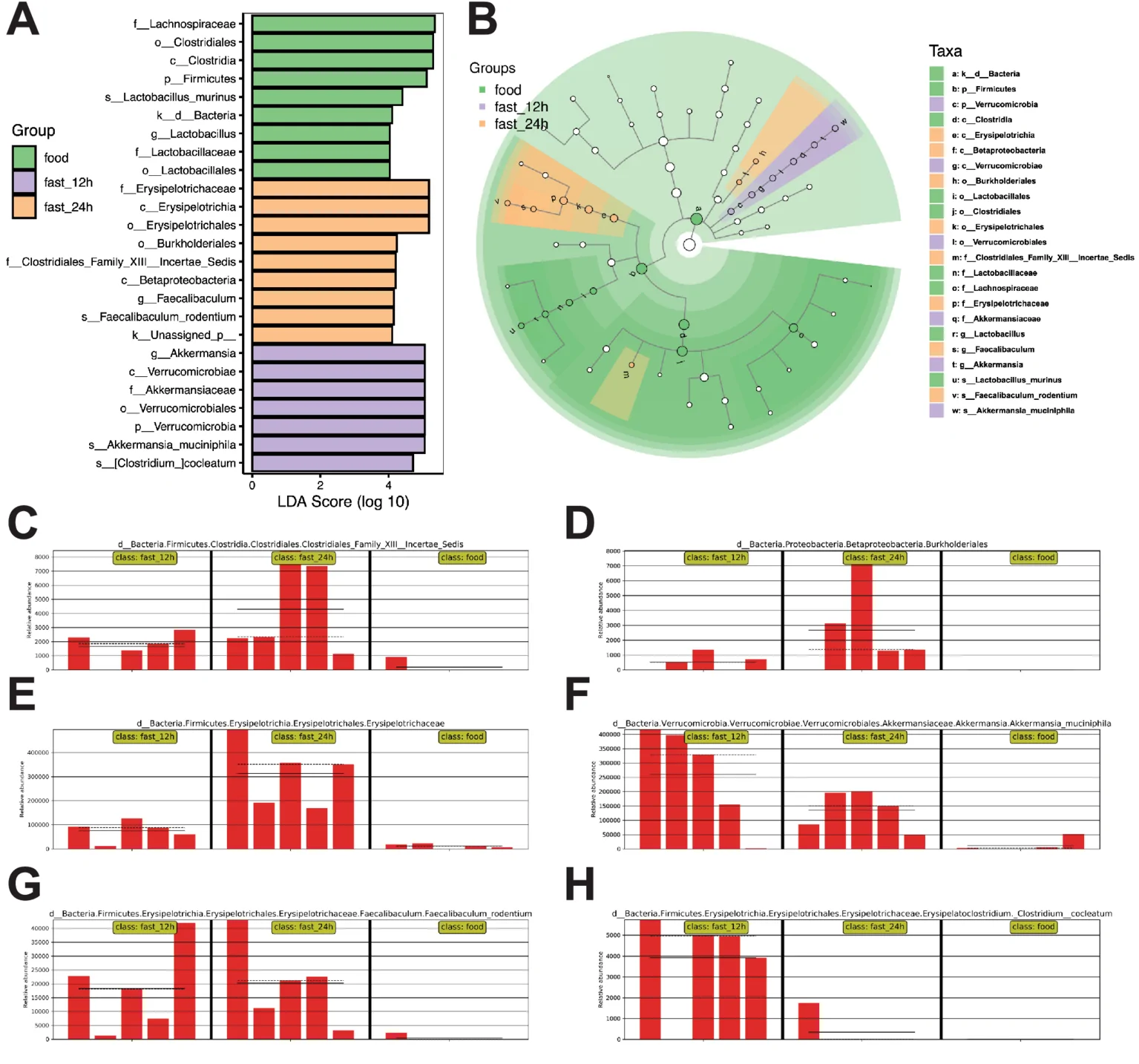
Fasting alters the gut microbiota composition in a time-dependent manner. **(A)** LDA score distribution of microbial taxa with significant differences across the three groups, identified by LEfSe analysis. A threshold of LDA score >2.0 and *p* < 0.05 was applied. **(B)** Cladogram depicting the taxonomic hierarchy of differential taxa. Circles represent different taxonomic levels, from phylum (innermost) to species (outermost), with colors indicating the group in which the taxa were enriched. **(C–H)** Bar plots illustrating the relative abundances of specific taxa that exhibited significant differences across the groups: **(C)** *Clostridiales Family XIII Incertae Sedis*, **(D)** *Burkholderiales*, **(E)** *Erysipelotrichaceae*, **(F)** *Akkermansia muciniphila*, **(G)** *Faecalibaculum rodentium*, and **(H)** *Erysipelatoclostridium clostridium cocleatum*. Each bar represents an individual mouse, and horizontal dashed lines indicate the mean abundance for each group.

Linear discriminant analysis effect size (LEfSe) identified several bacterial taxa with significant differences among the three groups (Figures 3A,B). The abundance of *Akkermansia muciniphila*, a mucin-degrading bacterium, was nearly undetectable in the ad libitum feeding group but significantly increased after 12 h of fasting (LDA score = 5.06, *p* = 0.0297), followed by a slight decrease at 24 h (Figure 3F). This species has been linked to gut barrier maintenance and metabolic health, suggesting a potential role in fasting-induced gut adaptation, which aligns with its increased prevalence in the fasting groups (Figure 2E).

*Lactobacillus murinus*, a species within Lactobacillus, exhibited a marked reduction at 12 h of fasting, followed by partial recovery at 24 h (LDA score = 4.40, *p* = 0.0132) (Figure 3A). A similar trend was observed in its relative abundance pattern (Figure 2E). Conversely, *Clostridiales Family XIII Incertae Sedis* displayed a progressive increase, reaching its highest abundance at 24 h (LDA score = 4.21, *p* = 0.0139) (Figure 3C).

*Erysipelatoclostridium clostridium cocleatum* was absent in the food group but significantly increased at 12 h of fasting (LDA score = 4.71, *p* = 0.0158), then sharply declined at 24 h (Figure 3H). Similarly, *Burkholderiales* (*Betaproteobacteria*) exhibited a moderate increase at 12 h and a more pronounced elevation at 24 h (LDA score = 4.21, *p* = 0.0272) (Figure 3D), which is notable given that some *Proteobacteria* species are associated with gut dysbiosis and inflammation.

At the family level, Erysipelotrichaceae also showed fasting-associated changes across the three groups (Figure 3E). Meanwhile, *Faecalibaculum rodentium* (*Erysipelotrichaceae* family) was significantly enriched in the 24 h fasting group (LDA score = 4.15, *p* = 0.0118) (Figure 3G), supporting the notion that extended fasting facilitates microbial shifts towards taxa involved in energy metabolism. This finding aligns with the observed changes in microbial abundance across fasting conditions (Figure 2E).

Overall, these findings demonstrate that fasting induces time-dependent remodeling of the gut microbiota, affecting microbial species associated with mucin degradation, immune regulation, and metabolic functions. The observed shifts in *Akkermansia*, *Lactobacillus*, and *Faecalibaculum* highlight the potential role of fasting in modulating gut microbial ecology, with possible implications for host metabolic adaptation.

### 3.4 Fasting induces metabolic shifts and pathway alterations

**Figure 4:**
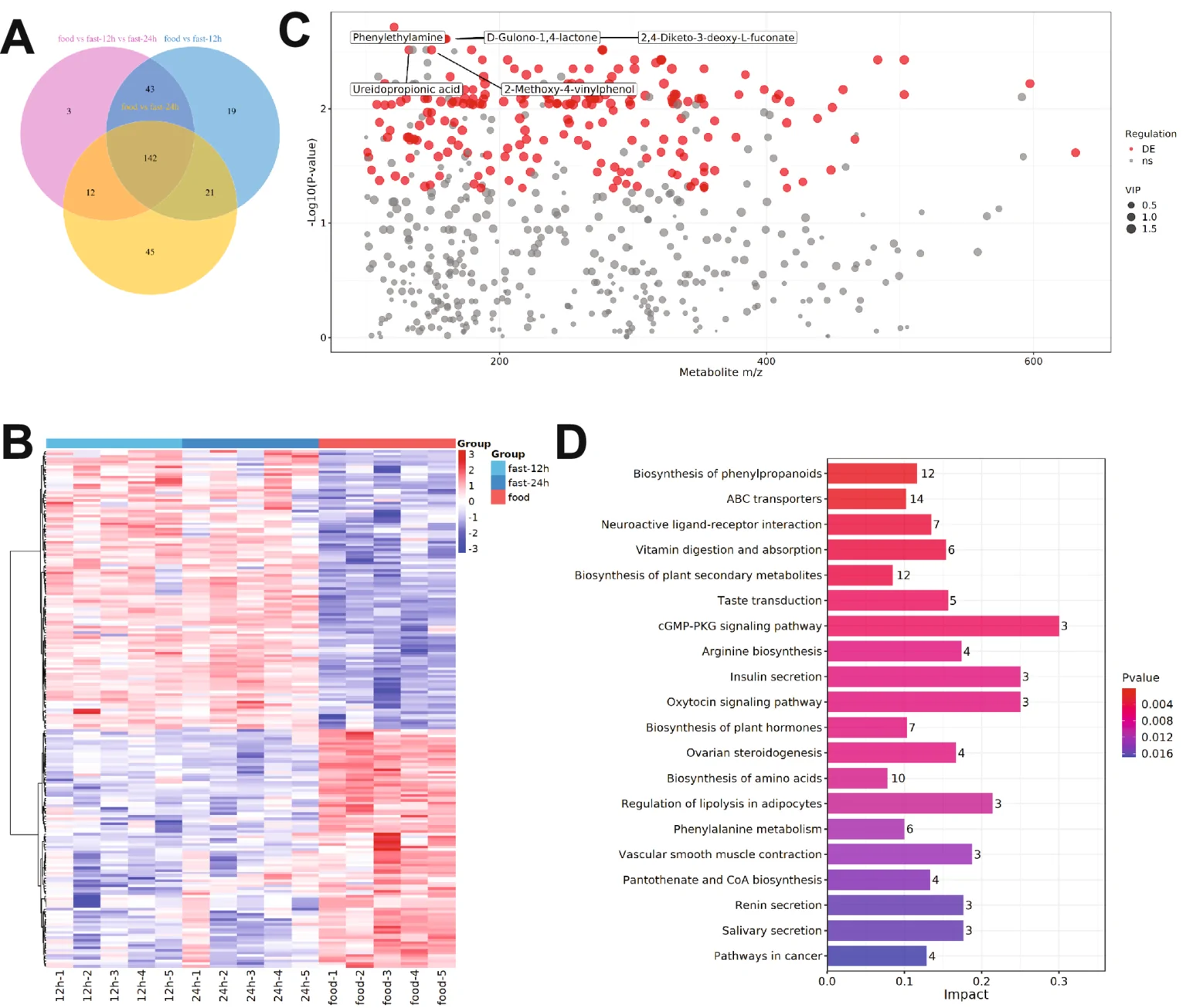
Fasting-induced metabolic alterations and pathway enrichment analysis **(A)** Venn diagram showing the overlap of differentially abundant metabolites among the Food, Fast_12h, and Fast_24h groups (*n* = 5) **(B)** Heatmap of significantly altered metabolites across groups, with hierarchical clustering based on Z-score-normalized intensities **(C)** Volcano plot illustrating the distribution of differential metabolites. Significant metabolites are labeled, with point size representing the VIP score **(D)** Pathway enrichment analysis displaying metabolic pathways associated with fasting-responsive metabolite changes.

Fasting led to significant changes in metabolic profiles, as reflected in the differential metabolite analysis. The Venn diagram (Figure 4A) illustrates the overlap of altered metabolites among the food, 12 h fasting, and 24 h fasting groups. A core set of 142 metabolites was commonly affected across all comparisons, indicating shared metabolic responses to fasting, while distinct sets of metabolites were uniquely modulated at different fasting durations.

The hierarchical clustering heatmap (Figure 4B) presents the relative abundance of significantly altered metabolites across groups. While fasting-induced metabolite fluctuations were observed at both 12 h and 24 h, the metabolic shifts became more pronounced with long-duration fasting. Notably, metabolites involved in amino acid metabolism, nucleotide metabolism, and lipid metabolism exhibited significant alterations, reflecting fasting-induced metabolic adjustments aimed at maintaining energy homeostasis.

The volcano plot (Figure 4C) highlights key differential metabolites based on their significance (−log10 *p*-value) and mass-to-charge ratio (m/z). Among them, phenylethylamine, D-Gulono-1,4-lactone, 2,4-diketo-3-deoxy-L-fuconate, ureidopropionic acid, and 2-methoxy-4-vinylphenol showed significant fasting-related variations, suggesting their involvement in adaptive metabolic responses.

Pathway enrichment analysis (Figure 4D) revealed several metabolic pathways that were significantly impacted by fasting. The most enriched pathways included phenylpropanoid biosynthesis, ABC transporters, neuroactive ligand-receptor interaction, and vitamin digestion and absorption, which are associated with neurotransmitter activity, microbial interactions, and energy metabolism. Additionally, pathways such as arginine biosynthesis, insulin secretion, and cGMP-PKG signaling were significantly altered, indicating potential regulatory effects on amino acid metabolism, glucose homeostasis, and vascular function.

The identified metabolic shifts and enriched pathways suggest systemic metabolic adaptations to fasting, encompassing amino acid metabolism, neurotransmitter-related compounds, and lipid-derived molecules. Given that many of these metabolic pathways are influenced by gut microbial metabolism, fasting-induced microbiota composition changes may partly explain the observed metabolic alterations. Further investigation into the gut microbiota-metabolite interplay could offer insights into how microbial communities influence host metabolic responses during fasting conditions.

### 3.5 Correlations between gut microbiota and key metabolites are associated with fasting-related inflammatory and metabolic changes

**Figure 5:**
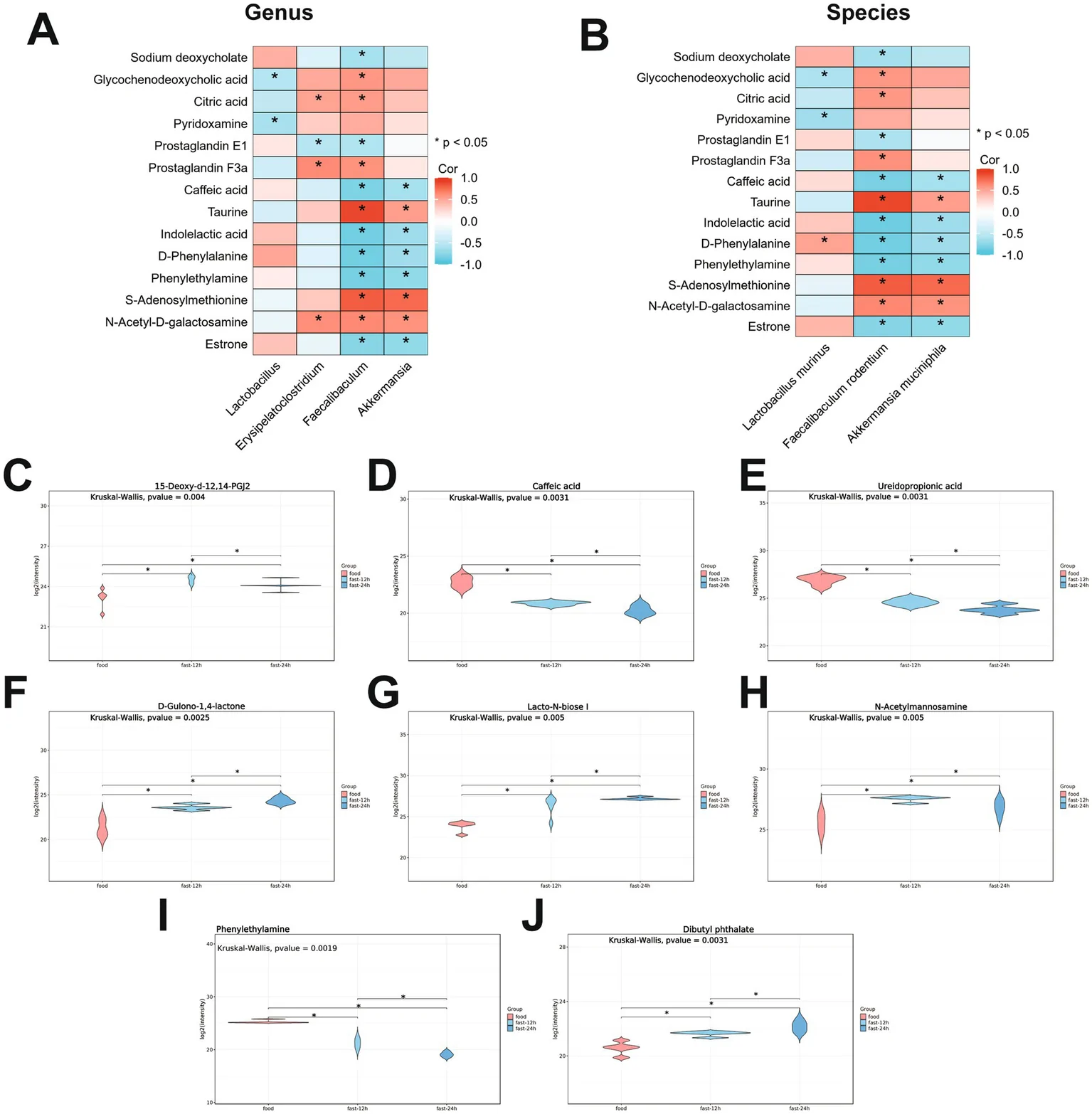
Correlation analysis reveals associations between fasting-altered gut microbiota and host metabolites. **(A,B)** Heatmaps display Spearman correlation coefficients between selected gut microbiota and metabolites at the genus **(A)** and species **(B)** levels (*n* = 5). Red indicates positive correlation, and blue indicates negative correlation. Significant correlations are marked with asterisks. **(C–J)** Violin plots showing the relative abundance of selected metabolites across the Food, Fast_12h, and Fast_24h groups (*n* = 5): **(C)** 15-Deoxy-d 12,14-PGJ2, **(D)** Caffeic acid, **(E)** Ureidopropionic acid, **(F)** D-Gulono-1,4-lactone, **(G)** Lacto-N-biose I, **(H)** N-Acetylmannosamine, **(I)** Phenylethylamine, and **(J)** Dibutyl phthalate.

The correlation analysis between gut microbiota and key metabolites revealed distinct associations between fasting-responsive microbial taxa and metabolic changes (Figures 5A,B). At the genus level, *Akkermansia* and *Faecalibaculum* exhibited significant correlations with metabolites involved in mucosal homeostasis, inflammatory regulation, and host metabolic responses (Figure 5A). Specifically, *Akkermansia* was positively associated with taurine (*p* < 0.05), whereas it was negatively associated with caffeic acid, indolelactic acid, and D-phenylalanine (*p* < 0.05). *Faecalibaculum* displayed similar correlation patterns with several metabolites, including phenylethylamine and S-adenosylmethionine (*p* < 0.05), suggesting fasting-related microbiota-metabolite coordination at the genus level.

At the species level, *Akkermansia muciniphila* and *Faecalibaculum rodentium* showed broadly consistent correlation patterns with their corresponding genera (Figure 5B). *Akkermansia muciniphila* was positively correlated with taurine and negatively correlated with caffeic acid, indolelactic acid, and D-phenylalanine (*p* < 0.05). These associations may reflect a barrier-related metabolic adaptation during fasting rather than direct evidence of microbial regulation of epithelial function.

*Erysipelatoclostridium* and *Erysipelatoclostridium clostridium cocleatum* exhibited significant correlations with inflammatory lipid mediators, including prostaglandin E1 and prostaglandin F3a (*p* < 0.05), suggesting an association with fasting-related inflammatory metabolic changes (Figures 5A,B). In addition, citric acid, a central component of the tricarboxylic acid cycle, was significantly correlated with *Erysipelatoclostridium*-related taxa (*p* < 0.05), implying a possible link between fasting-associated microbial shifts and altered energy metabolism. However, these associations do not establish whether the observed metabolites were derived from microbial metabolism, host metabolism, or both.

Further analysis of fasting-induced metabolite fluctuations revealed distinct trends among selected metabolites (Figures 5C–J). The levels of 15-Deoxy-d 12,14-PGJ2, caffeic acid, ureidopropionic acid, phenylethylamine, and dibutyl phthalate showed significant fasting-associated changes (Figures 5C–E,I,J; *p* < 0.05). In contrast, D-Gulono-1,4-lactone, Lacto-N-biose I, and N-Acetylmannosamine increased under fasting conditions (Figures 5F–H; *p* < 0.05), suggesting altered carbohydrate-, glycan-, or host substrate-related metabolism during fasting. These metabolite patterns further support the presence of duration-dependent metabolic remodeling in response to acute fasting.

Taken together, these correlation analyses indicate that fasting-responsive microbial taxa were statistically associated with metabolites involved in epithelial homeostasis, inflammation, and metabolic adaptation. In particular, *Akkermansia* and *Faecalibaculum* showed associations with metabolites related to mucosal homeostasis and host metabolic responses, whereas *Erysipelatoclostridium* was correlated with inflammatory lipid mediators. However, these findings should be interpreted as exploratory associations rather than evidence of direct microbial regulation of metabolite production or inflammatory responses.

### 3.6 Fasting-induced colonic morphological changes and increased susceptibility to inflammatory challenges

**Figure 6:**
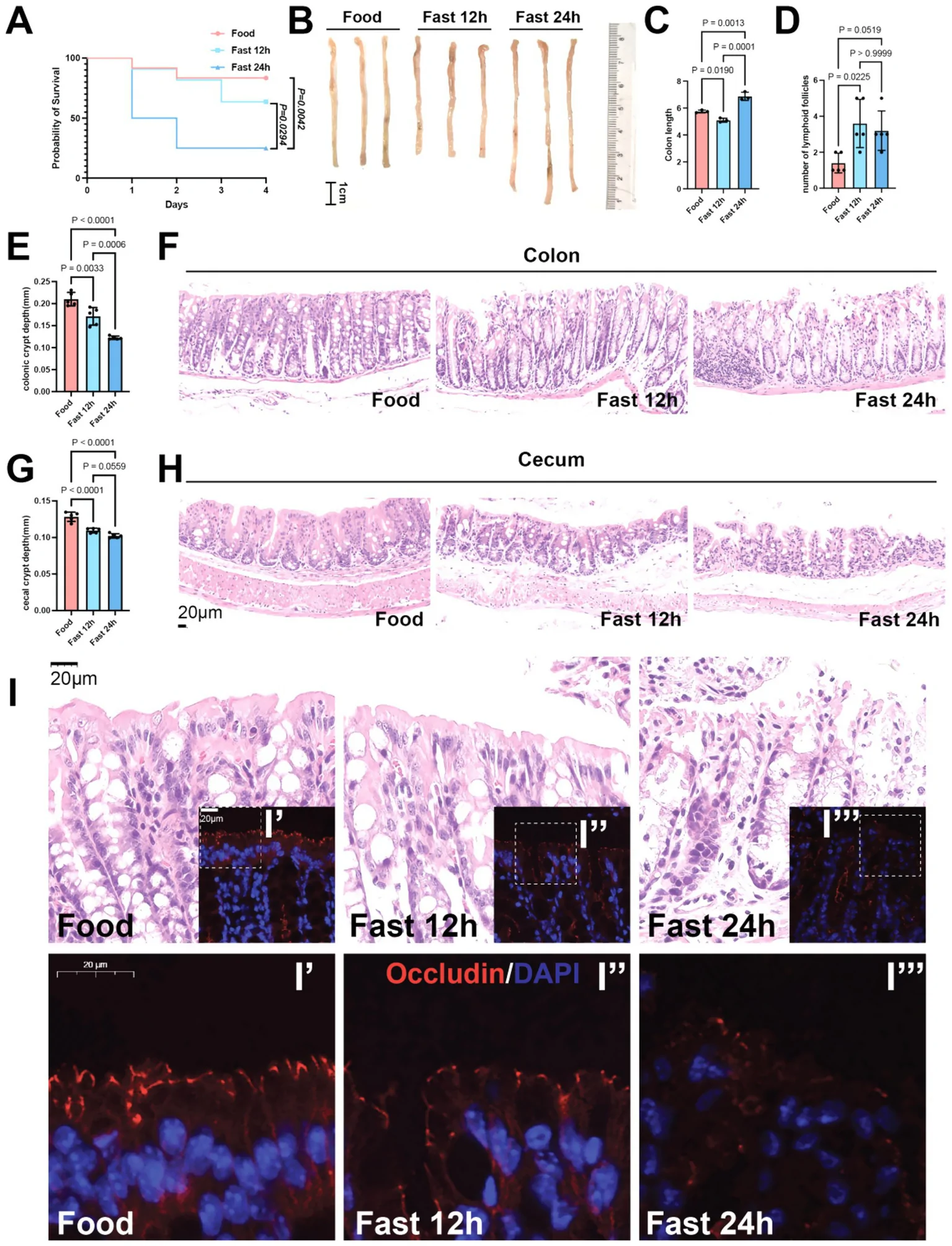
Fasting duration influences intestinal morphology and susceptibility to inflammatory stress. **(A)** Survival curves for CLP-induced sepsis in mice subjected to different fasting durations (*n* = 12). **(B,C)** Representative images and quantification of colon length from intact colon samples suitable for standardized gross measurement (*n* = 3). **(D)** Quantitative analysis of colonic lymphoid follicles after different fasting durations (*n* = 5). **(E,F)** Quantification and representative H&E-stained histological sections of colonic gland depth under various fasting conditions (*n* = 5). **(G,H)** Quantification and representative H&E-stained histological sections of cecal gland depth following fasting (*n* = 5). For histological quantification, five randomly selected non-overlapping microscopic fields were analyzed per mouse and averaged for each animal. **(I)** Representative histopathological analysis of colon mucosa and immunofluorescent staining of Occludin, with nuclei counterstained using DAPI. Scale bars represent 20 μm.

To further assess whether fasting-induced microbial and metabolic changes were accompanied by intestinal morphological alterations and altered inflammatory susceptibility, we examined colonic and cecal morphology and performed a CLP inflammatory challenge experiment. Survival analysis showed that 24 h fasting was associated with higher mortality after CLP compared with the Food and Fast_12h groups (Figure 6A, *p* = 0.0042 vs. Food; *p* = 0.0294 vs. Fast_12h). Because CLP is a severe polymicrobial inflammatory challenge, this result should be interpreted as reduced resilience to systemic inflammatory stress rather than direct evidence that mortality was caused solely by intestinal barrier dysfunction.

Gross morphological assessment showed fasting-associated changes in colon length among intact samples suitable for standardized measurement (Figures 6B,C). In addition, the number of zolonic lymphoid follicles tended to increase after fasting, suggesting possible changes in mucosal immune status (Figure 6D). Histological analyses of the colon and cecum revealed reduced gland depth after both 12 h and 24 h fasting, with more evident changes after 24 h (Figures 6E–H, *p* < 0.05 vs. Food). These findings indicate fasting-associated epithelial structural alterations rather than providing direct functional evidence of barrier impairment.

Immunofluorescent staining of Occludin further showed a reduced epithelial Occludin signal, particularly after 24 h fasting (Figures 6I′–I′′′). Together, these results suggest that fasting induced early barrier-associated structural changes, while prolonged fasting was associated with greater epithelial vulnerability and reduced resilience to severe inflammatory challenge.

## 4 Discussion

Fasting has gained increasing attention as a metabolic intervention, with proposed benefits ranging from insulin sensitization to lifespan extension. Yet much of this enthusiasm rests on relatively narrow time windows of investigation, and the assumption that “longer is better” has rarely been tested at the level of the gut barrier. Our data suggest that this assumption requires caution. By contrasting 12 h and 24 h fasting in the same genetic background, we observed that the same intervention can shift along a benefit–risk axis within a single day, with the gut microbiota and the epithelium responding on partially decoupled timescales. We interpret this as evidence that fasting is not a monotonic stimulus but a dose-dependent one, and that the gut is among the first organs to register the inflection point.

The expansion of *Akkermansia muciniphila* under fasting was one of the most consistent microbial signals in our dataset. This observation is biologically plausible because, in the absence of dietary polysaccharides, host-derived mucin may become an important carbon source in the colon, and *A. muciniphila* is well adapted to this niche (16, 17). However, this finding should not be interpreted as direct evidence that *Akkermansia* protects or damages the intestinal barrier in our model. Rather, it suggests that fasting creates a microbial ecological state that favors mucin-associated taxa. The positive correlation between *Akkermansia* and *taurine*, together with its inverse correlation with caffeic acid and indolelactic acid, may reflect a transient microbial-metabolic adaptation during early fasting. Whether this adaptation is protective, compensatory, or metabolically costly requires direct functional testing.

The *Erysipelatoclostridium* signal is more difficult to interpret and, in our view, deserves greater caution than is generally afforded to single-genus correlations. Members of this genus have been reported as both pro- and anti-inflammatory in different disease contexts, and our positive correlation with prostaglandin E1 and F3a is, strictly speaking, an association rather than a causal link. Still, the simultaneous enrichment of citric acid — a cytosolic fuel that, when accumulating extramitochondrially, can drive lipogenesis and acetylation-dependent inflammatory programs (18) — suggests a coherent metabolic backdrop in which long fasting may be associated with a low-grade inflammatory metabolic state. We cannot exclude that part of this signal originates from host cells rather than the microbiota, and 16S data alone cannot resolve this; metatranscriptomics or targeted prostaglandin quantification in colonic tissue would be needed to confirm a microbial origin. We therefore frame *Erysipelatoclostridium* as a candidate biomarker of fasting-related inflammatory drift, not as an established pathobiont.

A point we wish to emphasize, because it has been underappreciated in the fasting literature, is the dissociation between microbial “improvement” and structural “damage.” At 12 h, the microbiota looks favorable (*Akkermansia* up, *Lactobacillus murinus* preserved) yet crypt depth is already measurably reduced. At 24 h, microbial richness by Chao1 actually rises, but Occludin staining is patchy and survival after CLP was reduced. This temporal mismatch is biologically meaningful: it suggests that microbial composition is a leading, but not sufficient, indicator of mucosal health. Investigators relying solely on 16S endpoints may therefore overestimate the safety of extended fasting. The concomitant assessment of barrier proteins, crypt morphology, and a functional challenge (here, CLP) may provide a more balanced framework for evaluating fasting-related intestinal safety.

The CLP results require cautious interpretation. CLP is a severe polymicrobial sepsis model involving intestinal injury, systemic inflammation, immune dysregulation, metabolic stress, and altered physiological reserves. Therefore, the higher mortality observed in 24 h-fasted mice should not be attributed solely to intestinal barrier dysfunction. A more conservative interpretation is that prolonged fasting reduced the overall resilience of mice to severe inflammatory stress. This reduced resilience may involve combined contributions from barrier-associated epithelial changes, reduced metabolic reserves, fasting-induced immune modulation, dehydration-related stress, altered activity, and other systemic adaptations. Thus, the CLP experiment should be viewed as a stress-challenge readout rather than a direct functional assay of intestinal permeability. Future studies combining permeability assays, immune profiling, and metabolic reserve assessment will be required to define the relative contribution of these mechanisms.

The metabolite trajectories add another layer of complexity. Caffeic acid and phenylethylamine declined progressively with fasting duration, whereas D-Gulono-1,4-lactone and Lacto-N-biose I increased (19, 20). These changes may reflect a shift from dietary-substrate-associated metabolism toward greater reliance on endogenous or host-derived substrates during fasting. However, this interpretation remains hypothetical because untargeted metabolomics cannot determine whether these metabolites originated primarily from host cells, microbial metabolism, or diet-derived substrates. Similarly, although the increase in Lacto-N-biose I is consistent with altered glycan-related metabolism, we did not directly measure mucus thickness, goblet cell function, mucin turnover, or microbial glycan utilization. Therefore, the proposed link between fasting, host glycan availability, *Akkermansia* enrichment, and epithelial vulnerability should be regarded as a working hypothesis for future investigation.

Several limitations should temper our conclusions. First, we studied only male C57BL/6 J mice, and sex-dependent differences in fasting responses, gut microbiota composition, and epithelial physiology were not addressed. Second, the study focused on acute fasting time points of 12 h and 24 h and therefore cannot be directly extrapolated to chronic intermittent fasting or repeated time-restricted feeding regimens. Third, no formal *a priori* power calculation was performed, and some endpoints, particularly gross colon length, were based on a limited number of intact measurable samples. Fourth, although histological assessment and Occludin immunofluorescence provided barrier-associated structural information, we did not perform direct functional permeability assays, such as FITC-dextran translocation, transepithelial electrical resistance, circulating endotoxin measurement, or additional tight-junction markers such as ZO-1 and Claudin-1. Fifth, fasting-related physiological confounders, including body weight loss, dehydration, stress hormone responses, reduced activity, and altered energy expenditure, were not systematically measured and may have contributed to the microbiota, metabolomic, and CLP survival phenotypes. Sixth, the microbiota-metabolite analyses were correlation-based and should not be interpreted as causal; fecal microbiota transplantation, antibiotic depletion, germ-free or gnotobiotic models, and targeted microbial interventions will be needed to define causality. Seventh, LEfSe-derived microbial signatures were not externally validated using additional differential abundance approaches such as ANCOM-BC, DESeq2, or ALDEx2, and selected taxa or metabolites were not confirmed by qPCR or targeted metabolomics. Finally, although full-length 16S rRNA sequencing improves taxonomic resolution compared with short-read approaches, it cannot reliably distinguish strain-level differences in *A. muciniphila* or other taxa that may have distinct host effects.

**Figure 7:**
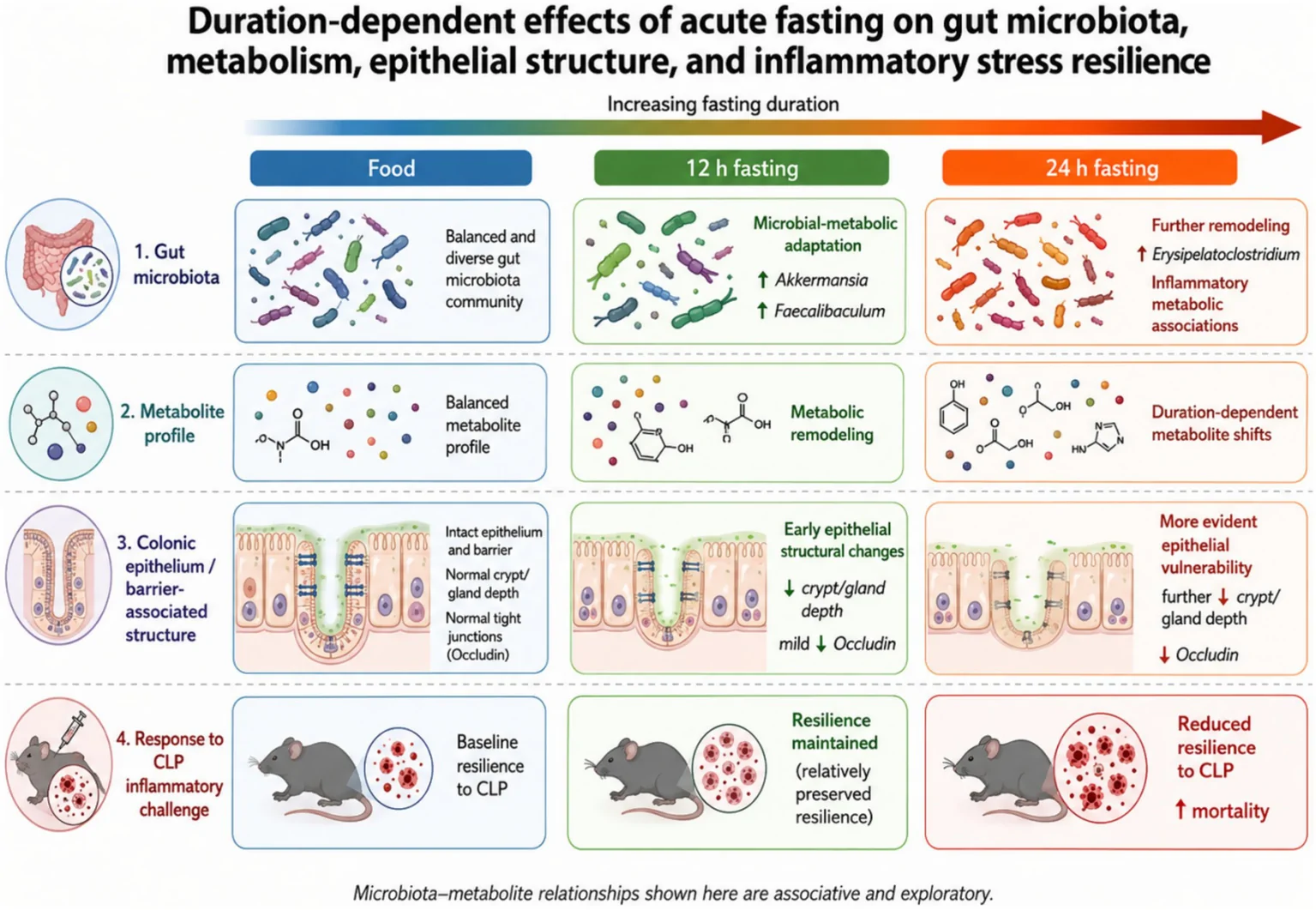
Schematic summary of the duration-dependent effects of acute fasting on gut microbiota, metabolism, epithelial structure, and inflammatory stress resilience. Under the food condition, mice showed a balanced gut microbiota, balanced metabolite profile, intact colonic epithelial structure, and baseline resilience to CLP inflammatory challenge. After 12 h fasting, microbial-metabolic adaptation was observed, including enrichment of *Akkermansia* and *Faecalibaculum*-related signatures, together with early epithelial structural changes such as reduced crypt/gland depth and mild reduction of Occludin signal. After 24 h fasting, further microbial and metabolic remodeling was accompanied by more evident epithelial vulnerability and reduced resilience to CLP-induced inflammatory stress, as reflected by increased mortality. The microbiota-metabolite relationships shown in the schematic should be interpreted as associative and exploratory rather than causal.

Taken together, our study suggests that fasting duration is a critical determinant of the balance between microbial-metabolic adaptation and epithelial vulnerability. Rather than demonstrating unequivocal barrier enhancement after short-term fasting, our data indicate that beneficial microbial signatures can coexist with early mucosal structural changes. Prolonged fasting was associated with more evident barrier-associated alterations and reduced survival under CLP-induced inflammatory stress. Together, these findings support a cautious, duration-sensitive view of fasting interventions, as summarized in Figure 7, and underscore the need for direct permeability measurements, mechanistic microbiota studies, and translational validation before extrapolating these results to human fasting strategies.

## Data Availability

The metabolomics data have been deposited to the MetaboLights repository (21) with the study identifier MTBLS15098.
