# Supplementary material for: Dual effects of fasting on gut microbiota and metabolism: protective adaptation versus barrier dysfunction risks
Source: Front Nutr. 2026 Aug 17;13:1895190. doi: 10.3389/fnut.2026.1895190 (PMC13528338; doi:10.3389/fnut.2026.1895190)
Supplement: Supplementary file 1 [file Table_1.docx]

**Supplementary Table S1.** PERMANOVA results for beta diversity based on weighted UniFrac, Bray-Curtis, and unweighted UniFrac distance matrices.

| A. Overall PERMANOVA | | | | |
| --- | --- | --- | --- | --- |
| Distance matrix | pseudo-F | R^2^ | *P* | |
| Weighted UniFrac | 5.086 | 0.459 | 0.001 | |
| Bray-Curtis | 2.596 | 0.302 | 0.001 | |
| Unweighted UniFrac | 2.496 | 0.294 | 0.001 | |
| B. Pairwise PERMANOVA | | | | |
| Distance matrix | Comparison | pseudo-F | *P* | *q* |
| Weighted UniFrac | Fast_12h vs Fast_24h | 2.967 | 0.019 | 0.019 |
| Weighted UniFrac | Fast_12h vs Food | 5.245 | 0.011 | 0.0165 |
| Weighted UniFrac | Fast_24h vs Food | 7.033 | 0.011 | 0.0165 |
| Unweighted UniFrac | Fast_12h vs Fast_24h | 1.419 | 0.093 | 0.093 |
| Unweighted UniFrac | Fast_12h vs Food | 2.412 | 0.014 | 0.021 |
| Unweighted UniFrac | Fast_24h vs Food | 3.58 | 0.012 | 0.021 |
| Bray-Curtis | Fast_12h vs Fast_24h | 1.926 | 0.031 | 0.031 |
| Bray-Curtis | Fast_12h vs Food | 2.363 | 0.019 | 0.0285 |
| Bray-Curtis | Fast_24h vs Food | 3.438 | 0.006 | 0.018 |
